# ID 228 - Monitoramento do Horizonte Tecnológico (MHT) de Inibidores de Janus Quinase (JAKi) para o Tratamento de Artrite Reumatoide

**DOI:** 10.5327/2237-9622.2025.v34s1.22

**Published:** 2025-11-25

**Authors:** Natália Dias Brandão, Luiza Costa, Renato Picoli, Renata Ferreira, Júlia Lima, Pedro Antônio Barbosa, Aline Rocha, Hellen Miyamoto

**Keywords:** JAKi, artrite reumatoide, monitoramento do horizonte tecnológico

## Abstract

**Introdução:**

Os medicamentos modificadores do curso da doença (MMCD), como os inibidores de Janus quinase (JAKi), têm sido essenciais no tratamento da artrite reumatoide (AR). Com o rápido progresso no desenvolvimento dos JAKi e a iminente aprovação regulatória, este MHT visa identificar os JAKi em desenvolvimento para o tratamento de AR.

**Método:**

Foram recuperados registros de ensaios clínicos nas bases ClinicalTrials.gov, WHO International Clinical Trials Registry e Cochrane Central Register of Controlled Trials. Foram utilizadas estratégias de busca com vocabulário controlado e sinônimos dos termos relacionados aos JAKi e à AR. Não foram aplicados filtros de restrição temporal, idioma ou status do estudo. Ensaios clínicos de fase 2 e 3 foram incluídos, enquanto estudos observacionais e tecnologias já aprovadas pela Agência Nacional de Vigilância Sanitária (Anvisa) foram excluídos.

**Resultados:**

Foram identificados cinco JAKi em desenvolvimento para o tratamento da AR. O peficitinibe, indicado para pacientes com AR que tiveram resposta inadequada a MMCD sintéticos convencionais (MMCDsc), como metotrexato, atua inibindo todos os membros da família JAK (JAK1, JAK2, JAK3 e TYK2). Esse medicamento foi avaliado nos ensaios clínicos NCT01649999, NCT01554696 e NCT01565655 (fase 2, completos e com resultados), além dos estudos NCT02308163 e NCT02305849 (fase 3, completos e com resultados). O decernotinibe também é destinado a pacientes que falharam aos MMCDsc. Trata-se de um inibidor seletivo de JAK3, avaliado nos ensaios NCT01052194 e NCT00441922 (ambos de fase 2, completos e com resultados). O CPL409116, por sua vez, é um inibidor de JAK/ROCK indicado para pacientes com AR que não responderam ao metotrexato e está atualmente em fase de recrutamento para o ensaio clínico NCT05374785 (fase 2). Já o TLL-018, um inibidor altamente seletivo de JAK1/TYK2, é indicado para pacientes com AR ativa que não responderam ao MMCDsc, tendo sido avaliado nos ensaios NCT05133297 (fase 2, completo, sem resultados) e NCT06020144 (fase 3, recrutando). Por fim, o itacitinibe é um inibidor seletivo de JAK1, indicado para pacientes com AR ativa, e foi analisado no ensaio clínico NCT01626573 (fase 2, completo, sem resultados).

**Conclusão:**

Os resultados indicam que os JAKi continuam a expandir seu papel no tratamento da AR. O peficitinibe e o decernotinibe já possuem resultados publicados. No entanto, embora os estudos de eficácia do peficitinibe tenham mostrado resultados satisfatórios, os ensaios de fase 3 foram realizados exclusivamente em pacientes asiáticos. O decernotinibe, por sua vez, apresentou eventos adversos (EAs) relevantes, e não há estudos clínicos em andamento. A maioria dos estudos foram controlados por placebo, apesar do registro de outros JAKi para a AR em diversos países. As demais tecnologias, CPL409116, TLL-018 e itacitinibe, ainda se encontram nas fases iniciais de investigação clínica. Esses achados reforçam o aumento de ensaios clínicos que investigam a eficácia e a segurança dos JAKi para pacientes com AR. No entanto, a futura aprovação do registro desses novos JAKi traz um desafio aos gestores de saúde, visto que esses medicamentos muitas vezes podem apresentar benefícios adicionais limitados, ou ainda agregar EAs importantes, sem gerar economia para os sistemas de saúde.

